# 2118. Comparative Analysis of Cefiderocol Mono- and Combination Therapy for Multi-Drug Resistant Gram-negative Infections

**DOI:** 10.1093/ofid/ofad500.1741

**Published:** 2023-11-27

**Authors:** Amer El Ghali, Ashlan Kunz-coyne, Kristen Lucas, Molly Tieman, Andrew Purdy, Gabriela Iturralde, Esther Garcia, Dana Holger, Suet-Ping Lau, Xhilda Xhemali, Michael P Veve, Michael J Rybak

**Affiliations:** University of New Mexico College of Pharmacy, Albuquerque, NewMexico; Anti-infective Research Lab, Wayne State University, Detroit, Michigan; Wayne State University, Detroit, Michigan; University of Indiana Health, Bloomington, Indiana; Indiana University Health, Bloomington, Indiana; Memorial Hospital West, Pembroke Pines, Florida; Memorial Hospital West, Pembroke Pines, Florida; Wayne State University, Detroit, Michigan; Orlando Health, Orlando, Florida; Cleveland Clinic, Cleveland, Ohio; Eugene Applebaum College of Pharmacy and Health Sciences, Detroit, Michigan; Eugene Applebaum College of Pharmacy and Health Sciences, Detroit, Michigan

## Abstract

**Background:**

Multi-drug resistant gram-negative infections are a growing concern, necessitating the development of novel treatments. Cefiderocol (CFDC) is a new siderophore cephalosporin that targets the iron transport mechanism used by many gram-negative pathogens. We aimed to compare the clinical outcomes of CFDC monotherapy and combination therapy in various infection types.

**Methods:**

We performed a retrospective cohort analysis at 5 academic medical centers from May 2022 to April 2023. Patients were included if they were ≥18 years old and received CFDC for ≥48 hours, excluding subsequent courses not separated by ≥90 days. The primary objective was composite clinical success (30-day survival, absence of 30-day infection recurrence and resolution of signs and symptoms), with secondary objectives including individual components of clinical success, on-treatment resistance, 30-day readmission, and adverse reactions. Descriptive statistics and logistic regression were used.

**Results:**

We analyzed 70 patients, with 35 in each treatment group. The cohort was predominantly male (64.3%), had a mean age of 55.5 years (17.6), and 67% were admitted to the Intensive Care Unit. The mean (SD) APACHE II scores were 12.8 (7.5) for monotherapy and 14.9 (6.7) for combination therapy (p=0.22), while the mean (SD) Charlson comorbidity scores were 5.0 (3.6) and 4.5 (3.5) for the respective groups (p=0.61). The most common infection source was respiratory tract (54.3%), with Pseudomonas aeruginosa (55.7%) and Acinetobacter baumannii (25.7%) as the most frequently isolated pathogens. The median (IQR) CFDC mean inhibitory concentration (MIC) was 0.5 µg/mL (0.25-1.0). The 30-day mortality rates for the monotherapy and combination groups were 20% and 28.6%, respectively. There was no significant difference in composite clinical success between the groups (p=0.59).

**Conclusion:**

Our preliminary data suggest that there is no difference in clinical success between CFDC monotherapy and combination therapy with another antibiotic. Further studies are needed to confirm these findings and evaluate the optimal use of CFDC in a variety of clinical settings.

**Disclosures:**

**Michael J. Rybak, PharmD, PhD, MPH**, Abbvie, Merck, Paratek, Shionogi, Entasis, La Jolla, T2 Biosystems: Advisor/Consultant

